# Dietary Marine Hydrolysate Improves Memory Performance and Social Behavior through Gut Microbiota Remodeling during Aging

**DOI:** 10.3390/foods12234199

**Published:** 2023-11-21

**Authors:** Camille Mougin, Mathilde Chataigner, Céline Lucas, Quentin Leyrolle, Véronique Pallet, Sophie Layé, Elodie Bouvret, Anne-Laure Dinel, Corinne Joffre

**Affiliations:** 1 Université Bordeaux, INRAE, Bordeaux INP, Nutrineuro, UMR 1286, 33076 Bordeaux, France; camille@abyss-ingredients.com (C.M.); mathilde@abyss-ingredients.com (M.C.); celine.lucas@inrae.fr (C.L.); quentin.leyrolle@inrae.fr (Q.L.); veronique.pallet@bordeaux-inp.fr (V.P.); sophie.laye@inrae.fr (S.L.); anne-laure.dinel@inrae.fr (A.-L.D.); 2 Abyss Ingredients, 56850 Caudan, France; elodie@abyss-ingredients.com; 3 NutriBrain Research and Technology Transfer, NutriNeuro, 33076 Bordeaux, France

**Keywords:** low-molecular-weight peptides, n-3 long-chain polyunsaturated fatty acids (n-3 LC-PUFAs), aging, social behavior, cognitive decline, stress response, memory, gut microbiota, marine functional food

## Abstract

Aging is characterized by a decline in social behavior and cognitive functions leading to a decrease in life quality. In a previous study, we show that a fish hydrolysate supplementation prevents age-related decline in spatial short-term memory and long-term memory and anxiety-like behavior and improves the stress response in aged mice. The aim of this study was to determine the effects of a fish hydrolysate enriched with EPA/DHA or not on the cognitive ability and social interaction during aging and the biological mechanisms involved. We showed for the first time that a fish hydrolysate enriched with EPA/DHA or not improved memory performance and preference for social novelty that were diminished by aging. These changes were associated with the modulation of the gut microbiota, normalization of corticosterone, and modulation of the expression of genes involved in the mitochondrial respiratory chain, circadian clock, neuroprotection, and antioxidant activity. Thus, these changes may contribute to the observed improvements in social behavior and memory and reinforced the innovative character of fish hydrolysate in the prevention of age-related impairments.

## 1. Introduction

Healthy aging has become one of the primary social and economic challenges of the 21st century for nations; particularly as the elderly population (e.g., 65 years and older) has tripled from 4% to 13% and it is projected to double in the next three decades, reaching 20% of the population in 2025 and 33% in 2050 [1,2]. Aging is characterized by a complex and dynamic remodeling of different neurobiological and physiological processes and behavioral changes. It is highly associated with increased susceptibility to cognitive impairments [3,4,5,6,7] and with a deficit in social cognition. Moreover, cognitive decline has an impact on social abilities [8]. And the maintenance of social interactions is known to be essential to increase quality of life and longevity [9,10]. More and more studies investigated the role of gut microbiota on cognition and social interaction across the lifespan [11,12] and particularly during aging. There is growing evidence that the gut microbiota actively participates in the aging process [13,14,15]. Age-related microbial dysbiosis is involved in reshaping immune responses during aging, which manifest as immunosenescence, inflammaging, and oxidative stress that accompany many age-associated alterations, like cognitive disorders [16,17]. Furthermore, disruptions in the microbiome–gut–brain axis could lead to the development of central nervous system pathologies [18,19,20,21] and behavioral disorders [12,22]. Notably, intestinal flora richness and diversity correlate with the extent of human social interactions, with reduced interactions causing decreased microbial diversity [23]. Moreover, targeting the microbiota during aging may be an effective strategy to prevent cognitive and social alterations.

Nutrition appears as an interesting innovative approach for preventing or delaying the development of age-related cognitive social behavior deficits by targeting microbiota [24]. Indeed, several studies reported the effects of nutrition on gut microbiota, notably peptides and omega-3 PUFAs [25,26,27,28,29,30]. We previously demonstrated that dietary supplementation with a fish hydrolysate containing low-molecular-weight peptides and n-3 LC-PUFAs effectively prevent age-related spatial short-term memory deficits and influence navigation strategies during spatial learning [31]. However, the impact of a fish hydrolysate containing low-molecular-weight peptides and n-3 LC-PUFA on social behavior through gut microbiota modulation remains unexplored. Understanding these aspects could offer valuable insights to better exploit the potential benefits of this dietary supplementation.

In this study, we investigated the potential effects of a fish hydrolysate supplementation, containing mainly low-molecular-weight peptides and n-3 LC-PUFAs, on the prevention of age-related social decline. To understand the mechanisms involved, we explored the microbiota as a platform that communicates with the brain through the gut–brain axis and that modifies the host metabolism. We then measured the stress response, expression of genes involved in the mitochondrial metabolism, circadian clock, inflammation, antioxidant defenses, beta-oxidation, and neuroprotection. We also explored whether the inclusion of n-3 LC-PUFAs to the existing fish hydrolysate could potentially have an additional effect.

## 2. Materials and Methods

### 2.1. Animals

Male C57Bl/6J mice, aged 7 weeks and 15 months, were obtained from Janvier Labs, Le Genest-Saint-Isle, France. They were reared in a standard 12 h light/12 h dark cycle, housed on cellulose bedding in a controlled environment (temperature maintained at 21–23 °C, humidity at 40%), and provided with ad libitum access to both food and water. All animal care and experimental procedures were conducted in compliance with the EU Directive 2010/63/EU for animal experiments and were approved by the national ethical committee overseeing the welfare and use of animals (approval ID A27756).

### 2.2. Diet

**Figure 1:**
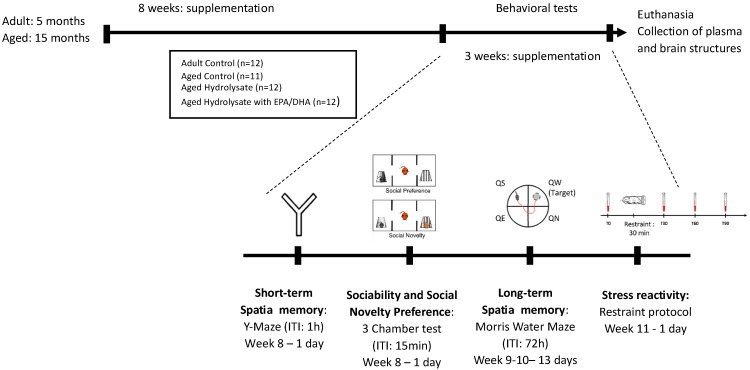
Experimental design. Adult (5 months) and aged (15 months) mice were fed the control diet or the hydrolysate-enriched diets for 8 weeks. Behavioral tests were performed during the next 3 weeks. Total supplementation duration was 11 weeks. DHA: docosahexaenoic acid; EPA: eicosapentaenoic acid; ITI: inter-trial interval; QE: quadrant east, QN: quadrant north; QS: quadrant south; QW: quadrant west.

After 11 weeks on a control diet, the mice were segregated into distinct groups: one group of adult mice (*n* = 12) and one of aged mice (*n* = 11) continued with the control diet. The two remaining groups of aged mice were provided with diets enriched with fish hydrolysate, comprising 0.21% of the hydrolysate for one group (*n* = 12) and 0.21% of the hydrolysate with an additional 0.09% of EPA/DHA for the other group (*n* = 12) (Table 1). Diets were provided by INRAE (Jouy en Josas, France). The diets were initiated when the adult mice were 5 months old and the aged mice were 15 months old, and these diets were maintained throughout the entire 11-week experiment (Figure 1). Consequently, by the end of the experiment, the adult and aged mice were approximately 8 and 18 months old, respectively. The fish hydrolysate, sourced from Abyss Ingredients in Caudan, France, was derived from marine byproducts and primarily consisted of low-molecular-weight peptides (<1000 Da) along with n-3 LC-PUFAs, like DHA and EPA. The detailed composition of the fish hydrolysate is outlined in patent number FR3099339(B1). The low-molecular-weight peptides dose (2.9 mg of low-molecular-weight peptides/mouse/day) corresponded to the quantity of peptides (<1000 Da) provided by 1 g of hydrolysate in humans and was determined by our previous results (not published). Polaris (Quimper, France) provided EPA (OMEGAVIE^®^ EPA 600 TG Qualitysilver^®^ 5) and DHA (OMEGAVIE^®^ DHA 70 TG). The dose of EPA/DHA was 1.56 mg/mouse/day (of which 1.248 mg/mouse/day of EPA and 0.312 µg/mouse/day of DHA) and corresponded to 250 mg/day of omega-3 in humans.

### 2.3. Behavioral Test

#### 2.3.1. Short-Term Spatial Recognition Memory Assessed in the Y-Maze

After eight weeks of supplementation, the Y-maze test was conducted to evaluate spatial recognition memory, following the procedure outlined by Dellu et al. [32]. The Y-shaped maze consisted of three arms, each 35 cm long and 10 cm deep, with a 15 lux illumination and extramaze visual cues on the walls to help mice orient themselves in the space. In the first trial, one arm of the Y-maze was closed, and mice were given 5 min to explore the remaining two arms. After an hour-long intertrial interval (ITI), mice were returned to the start arm for the second trial and given 5 min to explore all three arms. The start and closed arms were randomly assigned to each mouse, and their movements were tracked using video recording (SMART system; Bioseb, Vitrolles, France) to analyze the time spent in each arm. An index of recognition was computed to evaluate the animals’ performance against the expected chance level (33%).

#### 2.3.2. Spatial Memory Assessed in the Morris Water Maze Task

Spatial learning and memory were evaluated using the Morris water maze (MWM) test conducted following the method described in previous studies [33,34]. The test involved two days of familiarization, where mice had to locate a visible platform in a pool (3 trials/day, 60 s cut-off). This was followed by a day of cued learning to evaluate visuomotor deficits, where mice had to find a visible platform with the help of a cue (6 trials/day, 90 s cut-off). Spatial learning was then assessed over four consecutive days, during which mice were trained to find a submerged platform using distal extramaze cues (6 trials/day, 90 s cut-off). The Imetronic videotracking system was used to record the latency, distance traveled, and swim path for each trial. After 72 h, spatial memory was evaluated during a 60 s probe test in the maze without a platform.

The SMART system was utilized to measure the distance covered in each of the four quadrants, designating the quadrant where the platform was originally located during spatial learning as the target quadrant. Additionally, an index of recognition was computed to evaluate the animals’ performance against the expected chance level (25%).

#### 2.3.3. Examination of Navigation Strategies in the Morris Water Maze

**Figure 2:**
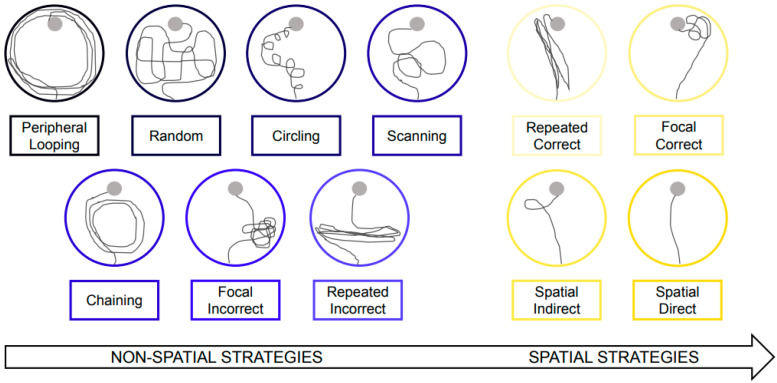
Categorization scheme of navigation paths. Representative navigation path patterns of “non-spatial” (in blue) and “spatial” (in yellow) strategies used to locate the platform.

For each trial in the spatial learning test, we evaluated the navigation path using the categorization system outlined by Bensalem et al. [32] (Figure 2). The navigation strategies were classified into two primary categories: nonspatial and spatial strategies. Nonspatial strategies encompassed initial “global search” strategies, such as “peripheral looping” and “random” along with “circling”. Additionally, there were “local search” strategies, including “scanning”, “chaining”, “repeated incorrect”, and “focal incorrect”. Spatial strategies, on the other hand, comprised “repeated correct”, “focal correct”, “spatial indirect”, and “spatial direct”.

#### 2.3.4. Sociability and Interest in Social Novelty in the Three-Chamber Test

To evaluate sociability and interest in social novelty, a three-chamber apparatus was used. The protocol was adapted from that described in Boyer et al. [35]. A rectangular arena (60 × 37.5 × 21 cm) made of transparent plexiglass plastic was divided into three compartments of the same size (18.5 × 37 × 21 cm). Two openings connected the center chamber with the two side chambers. A cylindrical container (10 cm in diameter, 20 cm high) was located in a corner of each side chamber. During the first trial (exploration), the target mouse was placed in the middle chamber facing a wall and allowed to explore. In the next trial (sociability), a stranger mouse was introduced in one of the containers, while the other was empty. In the last trial (social novelty preference), the same stranger mouse was introduced on one side, but a novel stranger was placed in the other container. The duration of each trial was 5 min, and animals were left for 15 min in their home cages between trials. Strangers were juvenile C57Bl/6J male mice (7 weeks) that were used to staying in the container. For each mouse, the stranger mice were randomly assigned to the left or right side of the chamber.

#### 2.3.5. Restraint Test to Explore Stress Reactivity

To evaluate their response to stress, mice were placed in a restraint device for 30 min. Blood samples were collected from the mandibular vein at 4 different time points: before the start of the restraint (T0), 30 min into the restraint (T30), 60 min into the restraint (T60), and finally via a transcardiac puncture at 90 min (T90).

### 2.4. Tissue Processing

Thirty minutes before the restraint test, the mice received an analgesic (buprenorphine, 300 mg/kg). After the 90 min restraint test, the mice were euthanized by an injection of exagon (300 mg/kg). The brain structures and plasma were collected and frozen at −80 °C until further analysis, following transcardiac perfusion with phosphate-buffered saline (PBS). Plasma was obtained by centrifuging the blood at 3000× *g* for 20 min.

### 2.5. Biochemical Measurements

#### 2.5.1. Measurement of Corticosterone

Plasma corticosterone levels were measured at four time points: before the onset of the stress protocol and 30, 60, and 90 min thereafter. An ELISA DetectX^®^ corticosterone immunoassay kit (Arbor assays, Strasbourg, France) was used for the measurements.

#### 2.5.2. Tryptophan (TRP) and Kynurenine (KYN) Levels

The concentrations of Trp and Kyn were measured with commercial ELISA kits following the instructions of the manufacturer (Trp: BAE-2700, Kyn: BAE-2200, ImmuSmol, Bordeaux, France). Optical density was measured using a microplate reader (Victor3V, PerkinElmer, Villebon-sur-Yvette, France) at a wavelength of 450 nm. Concentrations were calculated by reference to the standard curve.

#### 2.5.3. RNA Expression by Fluidigm Microfluidics Arrays

One microgram of total RNA was obtained from hippocampus as described in Dinel et al. [36] and was reverse-transcribed with SuperScript III reverse transcriptase (Invitrogen, Cergy-Pontoise, France). Diluted cDNA (2 µL, 5 ng/µL) was added to DNA Binding Dye Sample Loading Reagent (Fluidigm), EvaAreen (Interchim, Montluçon, France) and TE low EDTA to constitute Sample Mix plate. In Assay Mix plate, 10 µL of primer pairs (100 µM) was added to the Assay Loading Reagent (Fluidigm) and TE low EDTA to a final concentration of 5 µM. Following priming the chip in the Integrated Fluidic Circuit Controller, Sample Mix (5 µL) and Assay Mix (5 µL) were loaded into the sample inlet wells. One well was filled with water as a contamination control. To verify and specify target amplification and q-PCR process efficiencies, a sample control (mouse gDNA, Thermo Fisher, Waltham, MA, USA) was treated, preamplified, and quantified on assay control (RNaseP TaqMan probe, Thermo Fisher) using the same process in the same plate at the same time. The expected value of cycle quantification was around 13. The chip was placed into the IFC Controller, where 6.3 nL of Sample Mix and 0.7 nL of Assay Mix were mixed. Real-time PCR was performed on the Biomark System (Fluidigm) by BioControl to Grape platform (Bordeaux, France) with the following protocol: Thermal Mix at 50 °C, 2 min; 70 °C, 30 min; 25 °C, 10 min; Uracil-DNA N-glycosylase (DNA) at 50 °C, 2 min; Hot Start at 95 °C, 10 min; PCR cycle of 35 cycles at 95 °C, 15 s; 60 °C, 60 s; and melting curves (from 60 °C to 95 °C). Results were analyzed using the Fluidigm Real-time PCR Analysis software v.4.1.3. (San Francisco, CA, USA) to control specific amplification for each primer. Then, the raw data of the qPCR were analyzed using GenEx software version 7 (MultiD analyses AB, Freising, Germany) in order to choose the best reference gene to normalize mRNA expression and to measure the relative expression of each of the 93 genes analyzed between groups. The best reference gene was found to be β-2 microglobulin (B2m) that was thus used for normalization of gene expression.

#### 2.5.4. Analysis of the Microbiome in 16S

For DNA extraction we used Qiagen’s DNeasy 96 PowerSoil Pro Kit (Qiagen, Germany). Template DNA quantity in samples was determined by creating 1:8 and 1:64 dilutions of each sample and subjecting them all to qPCR using primers V5F_Nextera and V6R_Nextera targeting the V5V6 locus in the rRNA gene. Samples were diluted with water if necessary to normalize starting template abundances, and then amplicons were generated by subjecting sample DNA to 25-cycle PCRs using the same primers. PCR products were diluted 1:100 in water and subjected to a second 10-cycle PCR to attach Illumina sequencing primer-compatible DNA regions as well as individual barcodes for each sample. Samples were all uniquely dual-indexed, as detailed in Gohl, et al. [37]. Sequencing libraries were loaded onto an Illumina MiSeq using a 2 × 300 v3 flow cell (Illumina, San Diego, CA, USA). Sequenced data was analyzed using qiime2 [38]; taxonomy was assigned using the Silva release 132 database using 99% identities to the V5V6 16S region.

### 2.6. Statistical Analysis

Statistical analyses were conducted with GraphPad Prism 7 (GraphPadSotfware, San Diego, CA, USA). Heatmap was performed using the web interface of MetaboAnalyst (MetaboAnalyst 5.0, https://www.metaboanalyst.ca/MetaboAnalyst/home.xhtml (accessed on 15 February 2023), Canada).

A one-sample t-test was used to compare the four experimental groups against the expected chance level of 33% in the Y-maze and against the chance level of 25% in Probe test of MWM.

A 1-way ANOVA with diet as the factor was assessed to analyze the cued learning in the MWM, the time spent around the empty cage, the cage with stranger 1, the cage with stranger 2 in the 3-chamber test, and the gene expression. Fisher’s LSD post hoc tests were applied when appropriate.

A 2-way ANOVA with repeated measures was used to analyze the swim speed and spatial learning across training days in the MWM (factors: diet and days of learning) and the corticosterone levels (factors: diet and time of stress). Subsequently, Fisher’s LSD post hoc tests were conducted.

All data related to the gut microbiota were subjected to a 1-way ANOVA with diet as the factor. Post hoc Fisher’s LSD tests were performed when appropriate. Sequenced data were processed using the QIIME pipeline (v1.9.0) against the Greengenes (v13.8) database. Bioinformatic analyses and assessment of gut bacterial diversity (both beta and alpha diversity) were conducted using the web interface of MicrobiomeAnalyst (MicrobiomeAnalyst 2.0, https://www.microbiomeanalyst.ca/ (accessed on 10 June 2023), Canada).

All data were presented as means ± SEM. Differences were considered significant when the *p*-value was less than 0.05.

## 3. Results

### 3.1. Both Fish Hydrolysate Supplementations Promote Social Novelty Preference in Aged Mice

**Figure 3:**
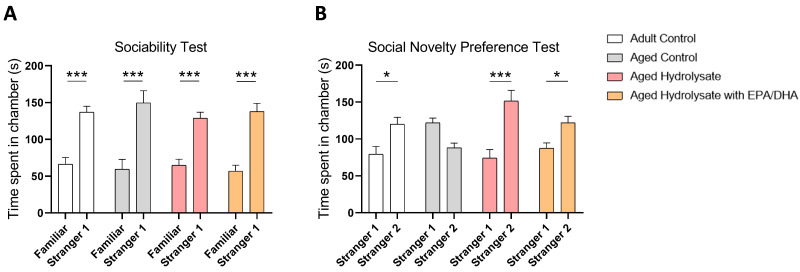
Both supplementations promote social novelty preference in aged mice. (**A**) Time spent around the empty cage and the cage with stranger 1. *** *p* < 0.001 Familiar vs. Stranger. (**B**) Time spent around the cage containing stranger 1 and the cage containing stranger 2. * *p* < 0.05, *** *p* < 0.001 Stranger 1 vs. Stranger 2. *n* = 11–12 per group.

We first evaluated social behavior in the three-chamber test. Each group of mice spent significantly more time exploring social target (stranger 1) than the empty cage (adult control, *p* < 0.001; aged control, *p* < 0.001; aged hydrolysate, *p* < 0.001; aged hydrolysate with EPA/DHA, *p* < 0.001) (Figure 3A) indicating that aging did not affect social interaction.

In the social novelty preference test, the adult control group spent significantly more time exploring the novel social target (stranger 2) compared to the stranger 1 (*p* = 0.02) (Figure 3B), which was not the case for the aged control group (*p* > 0.05). These observations reflected an impaired social recognition in aged mice. This was prevented in both supplemented groups (aged hydrolysate, *p* < 0.001; aged hydrolysate with EPA/DHA *p* = 0.05).

### 3.2. Both Fish Hydrolysate Supplementations Prevent Short-Term and Long-Term Spatial Memory Deficits in Aged Mice

The effect of the supplementations on short-term spatial memory was assessed using a Y-maze test with a 1 h ITI.

**Figure 4:**
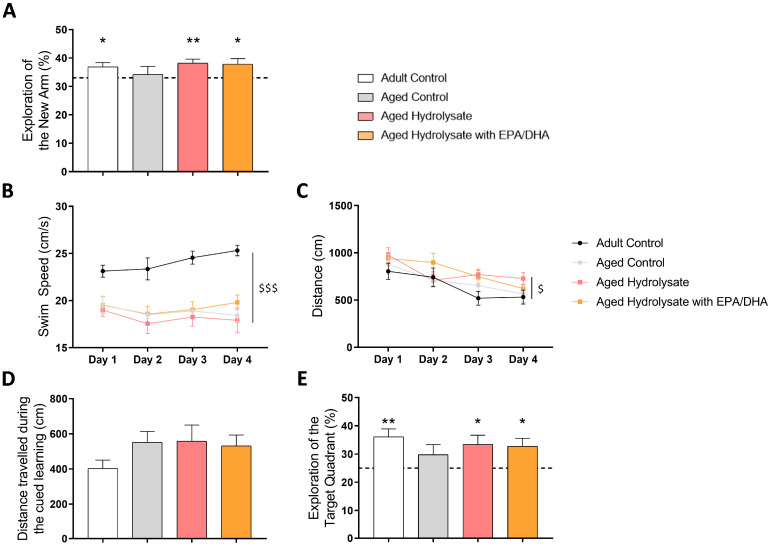
Both fish hydrolysate supplementations prevent short-term and long-term spatial memory deficits in aged mice. (**A**) Recognition index of the new arm after a 1 h ITI in adult and aged mice fed the control diet or the supplementations. The dotted line corresponds to chance level (33%) (* *p* < 0.05, ** *p* < 0.01 vs. chance level by one sample *t*-test). (**B**) Swim speed during learning (age effect: $$$ *p* < 0.001 by 2-way ANOVA with repeated measures). (**C**) Distance covered to reach the platform over the 4 consecutive days of spatial learning (day effect $ *p* < 0.05 by 2- way ANOVA with repeated measures). (**D**) Distance travelled during the cued learning. (**E**) Percentage of distance travelled in quadrants during the probe test. The dotted line represents chance level (25%) (* *p* < 0.05, ** *p* < 0.01 vs. chance level by one sample *t*-test). *n* = 11–12 per group.

The index of recognition of the adult control group was significantly higher than the chance level (33%), indicating an absence of spatial memory alterations (*p* = 0.03) (Figure 4A).

The aged control group presented a lack of recognition for the new arm, while the aged groups fed with supplementations spent significantly more time in this arm (aged hydrolysate, *p* = 0.004; aged hydrolysate with EPA/DHA, *p* = 0.03). Hence, the deficit observed in the aged control group was prevented by hydrolysate and hydrolysate with EPA/DHA supplementations.

Next, we evaluated the impact of the supplements on spatial learning and long-term memory using the Morris water maze. Due to a slower swimming speed in the aged control group compared to the adult control group (age effect, *p* < 0.001), we opted to measure the distance traveled to reach the platform as a more appropriate indicator of spatial learning acquisition (Figure 4B). All groups covered comparable distances to reach the platform, indicating comparable visual abilities (Figure 4C). Furthermore, all groups showed a significant reduction in distance covered over the four days of training (day effect, *p* = 0.02), indicating successful learning of the platform location (Figure 4D). Spatial memory was assessed 72 h after the final day of spatial learning. In comparison to the chance level of 25%, the adult control group covered more distance in the target quadrant than in the others (*p* = 0.002), whereas the aged control group did not show a significant difference (*p* > 0.05) (Figure 4E). These findings suggest a memory impairment in the aged control group, as the mice failed to recognize the target quadrant. Both supplementations effectively prevented this issue (aged hydrolysate, *p* = 0.03; aged hydrolysate with EPA/DHA, *p* = 0.02).

### 3.3. Fish Hydrolysate Supplementation Facilitates the Early Adoption of Spatial Strategies in the Process of Spatial Learning

**Figure 5:**
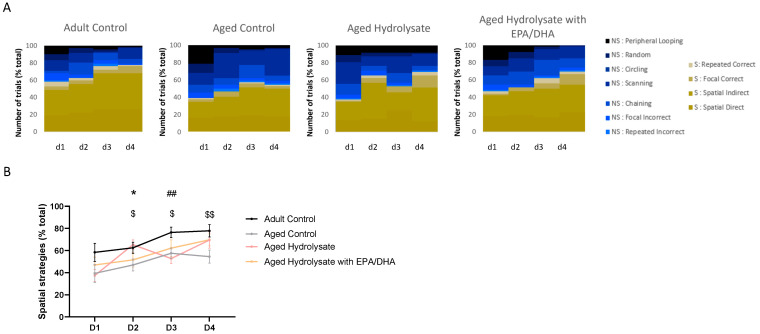
Fish hydrolysate supplementation promotes an earlier use of spatial strategies during spatial learning. (**A**) Navigational strategies used during spatial learning for each group. (**B**) Percentage of spatial strategies used by each group for each day of spatial learning. $ *p* < 0.05, $$ *p* < 0.01 Adult Control vs. Aged Control; ## *p* < 0.01 Adult Control vs. Aged Hydrolysate; * *p* < 0.05 Aged Hydrolysate vs. Aged Control, *n* = 11–13 per group. NS: non-spatial strategies; S: spatial strategies.

The examination of navigation patterns indicated a transition in strategies from nonspatial to spatial across the learning days for all mice in the four groups (Figure 5A). The application of spatial strategies showed a consistent increase after each day of training (time effect, *p* < 0.001) (Figure 5B). On the initial day of learning, all groups employed spatial strategies to a similar extent (*p* > 0.05). On the second day of training, the aged control group utilized fewer spatial strategies compared to the adult control group, indicating a deficiency in spatial learning (*p* = 0.04). This pattern persisted on day 3 and day 4 (day 3, *p* = 0.01; day 4, *p* = 0.009). On day 2, the aged hydrolysate group exhibited a restoration of spatial strategy usage to levels similar to the adult control group (*p* = 0.02), suggesting that the hydrolysate supplementation facilitates the use of spatial strategies. However, from day 3 to day 4 of training, the supplemented and the aged control groups were not significantly different (*p* > 0.05).

### 3.4. Both Supplementations Decrease Basal Stress and Modulate Stress Response in Aged Mice

**Figure 6:**
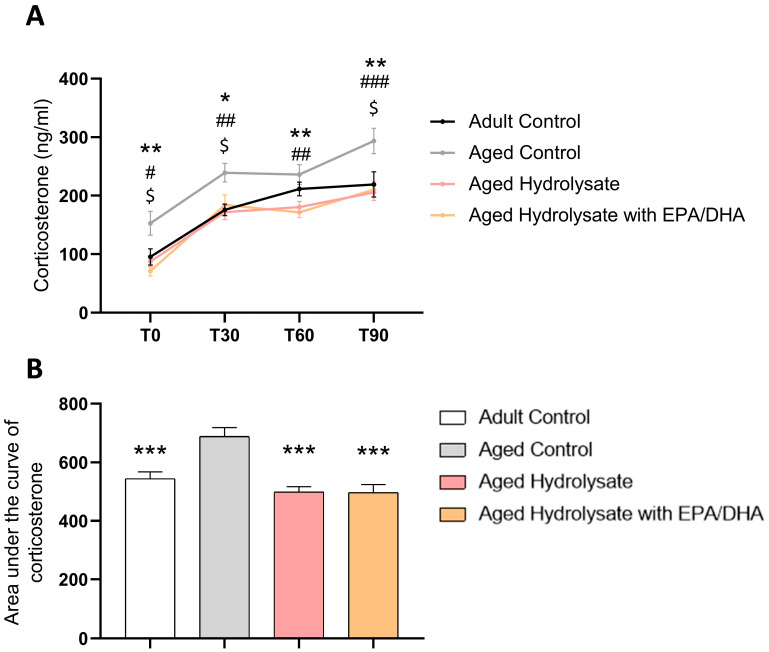
Both supplementations decrease basal stress and modulate stress response consecutive to restraint stress in aged mice. (**A**) Plasmatic corticosterone levels at T0, T30, T60 and T90. (**B**) Area under the curve of corticosterone. $ *p* < 0.05, Adult Control vs. Aged Control; # *p* < 0.05, ## *p* < 0.01, ### *p* < 0.001 Adult Hydrolysate vs. Aged Control; * *p* < 0.05, ** *p* < 0.01, *** *p* < 0.001 Aged Hydrolysate with EPA/DHA vs. Aged Control. *n* = 11–12 per group.

Plasma corticosterone levels were assessed in samples obtained prior to stress induction and at 30, 60, and 90 min after the initiation of the restraint protocol. The plasmatic corticosterone concentrations exhibited an elevation in response to the restraint protocol (time effect, *p* < 0.001) (Figure 6A).

The aged control mice displayed elevated levels of corticosterone before (T0, *p* = 0.03) and after stress (T30, *p* = 0.004; T90 *p* = 0.02) compared to the adult control mice and that was prevented by both supplementations (T0, aged hydrolysate, *p* = 0.01, aged hydrolysate with EPA/DHA, *p* = 0.003; T30, aged hydrolysate, *p* = 0.003, aged hydrolysate with EPA/DHA, *p* = 0.03); T60, aged hydrolysate, *p* = 0.01, aged hydrolysate with EPA/DHA, *p* = 0.01; T90, aged hydrolysate, *p* = 0.004, aged hydrolysate with EPA/DHA, *p* = 0.005).

The area under the curve (AUC) of corticosterone was significantly increased by age (*p* < 0.001), and this was rescued by both supplementations (aged hydrolysate, *p* < 0.001, aged hydrolysate with EPA/DHA, *p* < 0.001) (Figure 6B).

### 3.5. Fish Hydrolysate Supplementation Decreases the Levels of Plasmatic Kynurenine in Aged Mice

**Figure 7:**
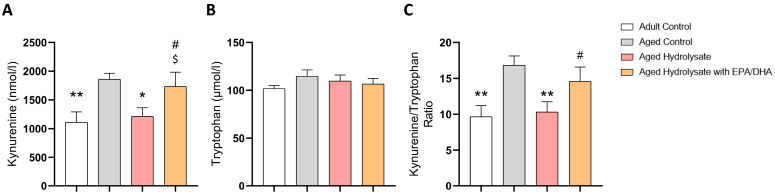
Fish hydrolysate supplementation decreases the levels of plasmatic kynurenine in aged mice. (**A**) Plasmatic levels of Kynurenine (**B**) Plasmatic levels of Tryptophan (**C**) Kynurenine/Tryptophan ratio. * *p* < 0.05, ** *p* < 0.01 vs. Aged Control; # *p* < 0.05 vs. Adult Control; $ *p* < 0.05 vs. Aged Hydrolysate. *n* = 11–12 per group.

The level of Kyn and the Kyn/Trp ratio were higher in the aged control mice than in the adult control mice (*p* = 0.006, *p* = 0.003, respectively) (Figure 7A,C). Interestingly, the Kyn level and the Kyn/Trp ratio were restored similar to the adult control’s level in aged hydrolysate mice (*p* = 0.02; *p* = 0.006, respectively) but not in the aged hydrolysate with EPA/DHA group. Age and supplementation had no effect on the level of Tryp (Figure 7B).

### 3.6. Both Supplementations Modulate Gene Expression Changes Associated with Aging

To understand the mechanisms involved in the prevention of social preference and memory deficits observed in the supplemented groups during aging, the expressions of 93 genes in the hippocampus were analyzed. These genes are involved in different pathways, such as inflammation, hypothalamic–pituitary–adrenal (HPA) axis, antioxidant defenses, heat shock protein response, neuroprotection, mitochondrial respiratory chain, circadian rhythm, β-oxidation, microglial phenotype, glucose metabolism, and synthesis of PUFAs and oxylipins. All genes that were significantly modulated are presented in Table 2.

**Figure 8:**
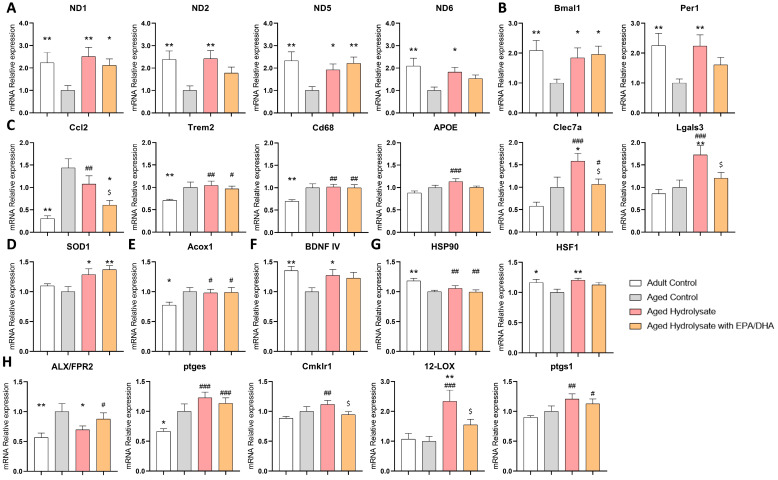
Both supplementations modulate gene expression changes associated with aging. (**A**) Mitochondrial respiratory Complex I. (**B**) Circadian clock. (**C**) Microglial neurodegenerative phenotype. (**D**) Antioxidant defenses. (**E**) beta-oxidation. (**F**) Neuroprotection. (**G**) Heat shock responses. (**H**) Oxylipins pathway. * *p* < 0.05, ** *p* < 0.01 vs. Aged Control; # *p* < 0.05, ## *p* < 0.01, ### *p* < 0.001 vs. Adult Control; $ *p* < 0.05 vs. Aged Hydrolysate. *n* = 11–12 per group.

The expression of *ND1*, *ND2*, *ND5,* and *ND6* involved in the formation of complex 1 of the mitochondrial respiratory chain was downregulated with age (Figure 8A). Interestingly, the expression of these four genes was significantly increased in the aged hydrolysate group compared to the aged group and restored to a similar expression level as the adult control group (*p* = 0.006; *p* = 0.004; *p* = 0.04; *p* = 0.02, respectively). In the same way, the expression level of *ND1* and *ND5* was similarly restored in the hydrolysate with EPA/DHA group compared to the adult control group (*p* = 0.04; *p* = 0.006, respectively).

The expression of *Bmal1* and *Per1* involved in the regulation of the circadian clock was also downregulated with age (*p* = 0.01; *p* = 0.008, respectively) (Figure 8B). The expression of *Bmal1* and *Per1* was significantly increased in the aged hydrolysate group to the same level as in the adult control group (*p* = 0.04; *p* = 0.008, respectively). The expression of *Bmal1* was significantly increased in the aged hydrolysate with EPA/DHA group as compared to the aged control group (*p* = 0.02).

The expression of six genes involved in microglial neurodegenerative phenotype was modulated (Figure 8C). The expression of *Ccl2*, *Trem2,* and *Cd68* was upregulated by age (*p* = 0.02; *p* = 0.003, *p* < 0.001, respectively). However, the expression of *Ccl2* significantly decreased in the aged hydrolysate with EPA/DHA group (*p* < 0.001). Both supplementations had no effect on the expression of *Trem2* and *Cd68* (*p* > 0.05). Moreover, the expression of *APOE*, *Clec7a*, and *Lgals3* was increased only in the aged hydrolysate group as compared to the adult control group (*p* < 0.001).

Although aging did not affect *SOD1* involved in antioxidant defenses, both supplementations increased its expression compared to the aged control group (aged hydrolysate, *p* = 0.01; aged hydrolysate with EPA/DHA, *p* = 0.002) (Figure 8D).

The expression of *Acox1* involved in β-oxidation was significantly increased in the aged control group as compared to the adult control group (*p* = 0.02). It was not modulated by the supplementations (*p* > 0.05) (Figure 8E).

*BDNF IV* expression was significantly decreased by age (*p* = 0.007) (Figure 8F). Interestingly, it was significantly increased by the hydrolysate supplementation at the same level as the adult control group (*p* = 0.04).

The expression of two genes involved in heat shock responses was modulated (Figure 8G). The expression of *HSP90* was significantly decreased in the aged control group as compared to the adult control and that was not restored by the supplementations (aged control, *p* = 0.002; aged hydrolysate, *p* = 0.02; aged hydrolysate with EPA/DHA, *p* = 0.001). The expression of *HSF1* was also significantly decreased by age (*p* = 0.02). The hydrolysate supplementation restored its expression at the same level as the adult control (*p* = 0.004).

The expression of four genes involved in the synthesis of oxylipins was modulated (Figure 8H). *ALX*/*FPR2* and *ptges* expression was significantly increased by age (*p* = 0.003; *p* = 0.02, respectively). Interestingly, *ALX*/*FPR2* expression was decreased in the aged hydrolysate group (*p* = 0.02). Hydrolysate with EPA/DHA supplementation had no effect on the expression of *ptges*. *Cmklr1*, *12-LOX,* and *ptgs1* expression was not affected by aging but was significantly upregulated in the aged hydrolysate group compared to the adult control (*p* = 0.007; *p* = 0.001; *p* = 0.005, respectively). The same result was obtained in the aged hydrolysate with EPA/DHA group for *ptgs1* expression (*p* = 0.04).

**Figure 9:**
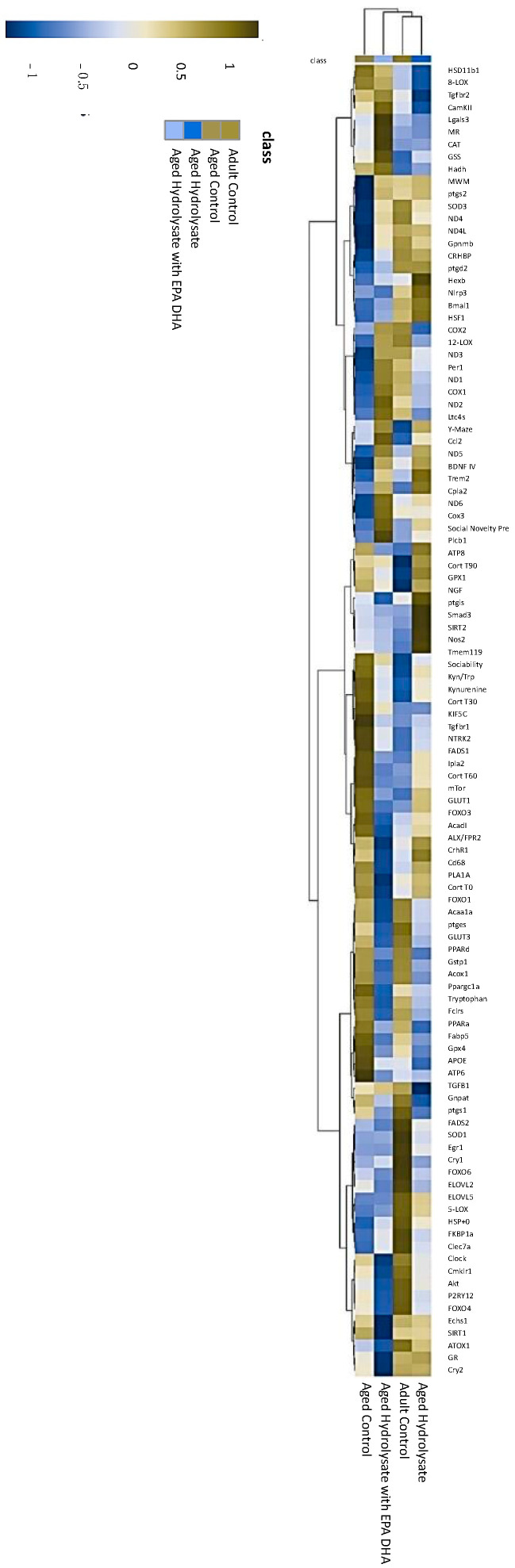
Heatmap analysis combined with hierarchical clustering analysis. Each row represents one gene and each column represents one group. Yellow indicates higher expression and blue indicates lower expression. The global gene expression profiles were compared after the end of 11-week supplementation, while aged control group were used for the baseline gene expression.

Finally, we performed a multivariate analysis. The result of a hierarchical clustering is displayed in a heat map as a dendrogram (Figure 9). Column dendrograms show the distance (or similarity) among the gene expression, behavioral results, and stress response. Row dendrograms show the distance (or similarity) between groups and which nodes each group belongs to as a result of the clustering calculation. Column dendrograms discriminated two clusters of parameters. The first cluster was composed of downregulated variables (blue color) in the aged control group compared to the adult control group. This cluster integrated genes from the mitochondrial respiratory chain (*ND1*, *ND2*, *ND3*, *ND4*, *ND4L*, *ND5*, *ND6*, *COX1*, and *COX2*), circadian clock (*Per1*, *Bmal1*), antioxidant defenses (*SOD3*), and behavioral variables that were altered in aged mice (social novelty preference, MWM and Y-maze). The second cluster was composed of upregulated parameters (red color) in the aged control group compared to the adult control: the stress response (corticosterone level), levels of kynurenine and tryptophan, and Kyn/Trp ratio. Then, row dendrograms separated the aged control group and the adult control group into two different clusters. Column dendrograms confirmed similarity between the adult control group and both supplemented groups. Indeed, both supplementations restored social novelty preference, MWM, and Y-maze parameters at the same level as in the adult control group. They also restored parameters from the stress response (corticosterone level), levels of Kyn and Trp, and Kyn/Trp ratio. From these results, both the aged-supplemented group and the adult control group were gathered in the same cluster. Further in the classification, the aged hydrolysate group is gathered with the adult control group suggesting a stronger similarity between these two groups.

### 3.7. Age and Supplementations Have Distinct Microbiota Profiles

**Figure 10:**
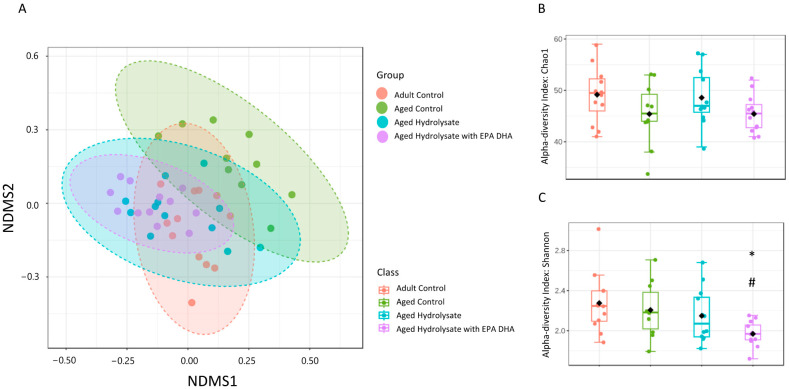
Microbial diversity analysis. (**A**) NMDS (non-metric multidimensional scaling). The closer the distance, the higher is the similarity. (**B**) Chao1 index. (**C**) Shannon index. * *p* < 0.05 vs. Aged Control; # *p* < 0.05 vs. Adult Control. *n* = 11–12 per group.

The beta diversity of the samples was analyzed using a nonmetric multidimensional scaling (NMDS) based on the binary Jaccard algorithm. The results suggest that there were significant differences in the community among the four groups (Figure 10A). Moreover, the intergroup distance was significantly greater than the intragroup distance (R = 0.18, *p* = 0.001), indicating that the four groups had distinct gut microbiota profiles.

Age and supplementations did not impact the species richness (Figure 10B). The Shannon index was significantly lower in the aged hydrolysate with EPA/DHA group compared to the adult control group (*p* = 0.02) and the aged control group (*p* = 0.04) (Figure 10C).

### 3.8. Both Supplementations Prevent Microbiota Impairments in Aged Mice

**Figure 11:**
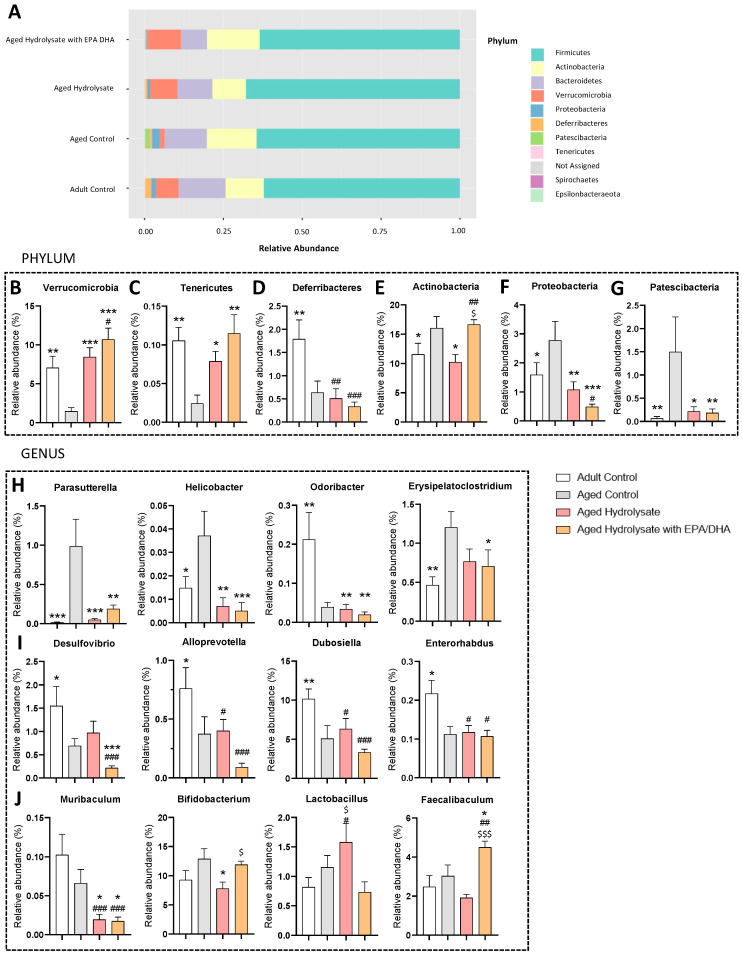
Both fish hydrolysate supplementations prevent microbiota impairments in aged mice. (**A**) Relative abundance of gut microbiota at phylum level. Relative abundance of the phyla (**B**) Verrucomicrobia. (**C**) Tenericutes. (**D**) Deferribacteres. (**E**) Actinobacteria. (**F**) Proteobacteria. (**G**) Patescibacteria. Relative abundance of gut microbiota at genus level. (**H**) *Parasutterella*, *Helicobacter*, *Odoribacter* and *Erysipelatoclostridium*. (**I**) *Desulfovibrio*, *Alloprevotella*, *Dubosiella* and *Enterorhabdus* (**J**) *Muribaculum*, *Bifidobacterium*, *Lactobacillus* and *Faecalibaculum*. * *p* < 0.05, ** *p* < 0.01, *** *p* < 0.001 vs. Aged Control; # *p* < 0.05, ## *p* < 0.01, ### *p* < 0.001 vs. Adult Control; $ *p* < 0.05, $$$ *p* < 0.001 vs. Aged Hydrolysate. *n* = 11–12 per group.

We analyzed the relative abundance of gut microbiota at the phylum level (Figure 11A). Of the 10 phyla analyzed, six were modulated by age and/or supplementations.

Verrucomicrobia (Figure 11B) and Tenericutes (Figure 11C) relative abundance was significantly decreased by age (*p* = 0.005; *p* = 0.003, respectively), and this was reversed by both supplementations (Verrucomicrobia, aged hydrolysate, *p* < 0.001, aged hydrolysate with EPA/DHA, *p* < 0.001; Tenericutes, aged hydrolysate, *p* = 0.04, aged hydrolysate with EPA/DHA, *p* = 0.001). Deferribacteres abundance was significantly decreased in the aged control group and in both supplemented groups as compared to the adult control group (aged control, *p* = 0.006; aged hydrolysate, *p* = 0.002; aged hydrolysate with EPA/DHA, *p* < 0.001) (Figure 11D).

Actinobacteria (Figure 11E), Proteobacteria (Figure 11F), and Patescibacteria (Figure 11G) relative abundance was significantly increased by age (*p* = 0.05; *p* = 0.04, *p* = 0.005 respectively), while both supplementations reached the levels of Patescibacteria and Proteobacteria (Patescibacteria, aged hydrolysate, *p* = 0.01, aged hydrolysate with EPA/DHA, *p* = 0.01; Proteobacteria, aged hydrolysate, *p* = 0.004, aged hydrolysate with EPA/DHA, *p* < 0.001). Actinobacteria abundance was restored only in the aged hydrolysate group at the same level as in the adult control group (*p* = 0.01).

At genus level, we observed seven genera modulated by age and eight by the supplementations. Age increased the relative abundance of *Parasutterella* (*p* < 0.001), *Helicobacter* (*p* = 0.01), *Odoribacter* (*p* = 0.003), and *Erysipelatoclostridum* (*p* = 0.005) (Figure 11H) that was reversed by both supplementations to the same level as the adult control group (*Parasutterella*, aged hydrolysate, *p* < 0.001, aged hydrolysate with EPA/DHA, *p* = 0.001; *Helicobacter*, aged hydrolysate, *p* = 0.001, aged hydrolysate with EPA/DHA, *p* < 0.001; *Odoribacter*, aged hydrolysate, *p* = 0.001, aged hydrolysate with EPA/DHA, *p* = 0.002). Only the aged hydrolysate with EPA/DHA group restored the relative abundance of *Erysipelatoclostridum* to the same level as the adult control (*p* = 0.04)

Age decreased the relative abundance of *Desulfovibrio* (*p* = 0.04), *Alloprevotella* (*p* = 0.04), *Dubosiella* (*p* = 0.002), and *Enterorhabdus* (*p* = 0.03) (Figure 11I). The decrease in *Desulfovibrio* relative abundance was more important in the aged hydrolysate with EPA/DHA group compared to the adult control group (*p* < 0.001). No effect of supplementations was found on the relative abundance of *Alloprevotella*, *Dubosiella,* and *Enterorhabdus*.

No effect of aging was observed for the relative abundance of *Muribaculum*, *Bifidobacterium*, *Lactobacillus,* and *Faecalibaculum* (Figure 11J). However, the relative abundance of *Muribaculum* was significantly decreased in both supplemented groups as compared to the adult control group (aged hydrolysate, *p* < 0.001; aged hydrolysate with EPA/DHA, *p* < 0.001); and aged control group (aged hydrolysate, *p* = 0.05; aged hydrolysate with EPA/DHA, *p* = 0.04) (Figure 11J). The relative abundance of *Bifidobacterium* was decreased, while *Lactobacillus* abundance was increased in the aged hydrolysate group compared to the aged control group (*Bifidobacterium*, *p* = 0.01; *Lactobacillus*, *p* = 0.03). The relative abundance of *Faecalibaculum* was increased in the aged hydrolysate with EPA/DHA group compared to the adult control group (*p* = 0.002) and the aged control group (*p* = 0.001).

### 3.9. Preference for Social Novelty and Memory Performance Correlates with AUC of Corticosterone, Plasmatic Level of Kyn, Kyn/Trp Ratio, Gene Expression of Complex 1 of the Mitochondrial Respiratory Chain, and Gut Microbiota Composition

The index of social novelty preference was negatively correlated with the AUC of corticosterone, level of Kyn, Kyn/Trp ratio, and relative abundance of *Erysipelatoclostridum* (AUC of corticosterone, r = −0.43, *p* = 0.003; Kyn, r = −0.47, *p* < 0.001; Kyn/Trp ratio, r = −0.45, *p* = 0.002; *Erysipelatoclostridum*, r = −0.36, *p* = 0.02). Conversely, the index of social novelty preference was positively correlated with the expression of *ND1*, *ND3*, *ND4*, and *ND6* and relative abundance of Verrucomicrobia (*ND1*, r = 0.30, *p* = 0.04; *ND3*, r = 0.32, *p* = 0.03; *ND4*, r = 0.31, *p* = 0.03; *ND6*, r = 0.28, *p* = 0.05; Verrucomicrobia, r = 0.38, *p* = 0.001). Moreover, the MWM index of recognition was positively correlated with the relative abundance of Verrucomicrobia (r = 0.38, *p* = 0.01). In contrast, the MWM index of recognition was negatively correlated with the relative abondance of Firmicutes (r = −0.42, *p* = 0.004) (Supplementary Materials Figure S1).

## 4. Discussion

As we previously demonstrated, supplementation with the fish hydrolysate prevented the age-related decline in spatial short-term memory and long-term memory and promoted an earlier use of spatial strategies in aged mice [31]. Here, we demonstrated that the same effect can be achieved with a lower dose of fish hydrolysate enriched with EPA/DHA or not.

The main finding of this study is that the fish hydrolysate and the fish hydrolysate enriched with EPA/DHA had a positive impact on age-induced social behavior alterations. Specifically, both supplementations improved preference for social novelty that was diminished by aging. These changes were associated with modulation of the gut microbiota that target different pathways, such as the stress response via the normalization of corticosterone levels, mitochondrial homeostasis via the modulation of the expression of genes involved in the mitochondrial respiratory chain, or the circadian rhythms. To our knowledge, our study is the first to evaluate the effect of a fish hydrolysate on sociability and social novelty preference. Recent research has shown that social behavior and relationships can change across the lifespan, a phenomenon referred to as ‘social aging’ [39,40]. Considering the significance of social integration for overall health and well-being, alterations in social behavior with age can influence how physical condition evolves over time. Our findings indicate that it was not the social interest that was altered during aging, but it was the social novelty preference, which confirmed recent reports [41,42]. Indeed, aged mice exhibited no preference for a novel foreign mouse. This loss of preference for social novelty may suggest a reduced ability to distinguish between individual mice, implying a social recognition deficit in aged mice. We demonstrated for the first time that fish hydrolysate supplementation can prevent the age-related effects on social novelty preference.

Understanding the molecular mechanisms involved in social preference and memory deficits is important to define the efficacy of fish hydrolysate supplementations to prevent age-related diseases. As (1) gut microbiota composition is modified during aging, (2) microbiota influence metabolism, and (3) communication axes exist between the gut microbiota and the brain, we investigated if the gut microbiota could be the hub that can explain behavioral changes during aging. We first analyzed the gut microbiota profile. Indeed, the gut microbiota play a crucial role in supporting healthy cognition and neurological functions of its host, including active participation in important aspects of brain aging. Gut microbial diversity decreases with aging [16,43,44,45,46]. This change has been associated with cognitive decline and social disturbances [21,47]. In our study, an NMDS analysis demonstrated that the gut flora profile of aged mice deviated from that of adult mice. Interestingly, we showed for the first time that fish hydrolysate supplementation restored the profiles of the intestinal flora in a way similar to the adult control group. Moreover, this restoration was associated with a rescue of cognitive and social behavior. Little is known about the effects of low-molecular-weight peptides present in the fish hydrolysate on microbiota, although a study reported that supplementation with small peptides from skipjack byproducts increases the diversity of the intestinal flora, which is in accordance with our results [48]. It should be interesting to analyze the metabolome induced by fish hydrolysate supplementations to better understand the observed effects.

Previous studies highlight a decline in bacteria phyla with age [16,43,44,45,46]. Compared with the control group, the abundance of Verrucomicrobia and Tenericutes was decreased in the aged group, while it was restored by both supplementations. The Spearman’s rank correlation analysis revealed that the level of Verrucomicrobia was positively correlated with the index of social novelty preference and the index of MWM recognition. A recent study reports that a lower abundance of Tenericutes has been observed in patients with cognitive impairment [49]. In addition, in healthy older adults, a positive correlation is found between the level of Verrucomicrobia and cognitive functions [50]. It has been reported that increased levels of Verrucomicrobia can suppress neurodegeneration [51] and reverse cognitive dysfunction, including improvements in impaired spatial working memory and recognition of new objects, while also restoring brain metabolism [52]. In various studies exploring cognition and gut microbiota in older adults, Verrucomicrobia have also been associated with better performance in tasks related to psychomotor processing speed, cognitive flexibility, and learning [50,53,54]. This suggests that the increase in both Verrucomicrobia and Tenericutes, induced by fish hydrolysate supplementations, can contribute to healthy aging by preventing cognitive dysfunction. Furthermore, we showed an increase in relative abundance of Proteobacteria in aged mice compared with the control group. This was rescued by both fish hydrolysate and fish hydrolysate with EPA + DHA supplementations. Proteobacteria, a major phylum of Gram-negative bacteria, are more abundant in older adults compared to younger adults. In previous studies, they have also been linked to increased gut inflammation and dysbiosis [55] and are negatively associated with executive function, learning, and memory [54].

At the genus level, our analysis revealed a significant increase in *Parasutterella*, *Helicobacter*, *Odoribacter,* and *Erysipelatoclostridium* in aged mice that was prevented by both supplementations. This result suggests that fish hydrolysate supplementations prevented dysbiosis observed during aging and could contribute to prevent cognitive dysfunction. Indeed, it has been reported that higher relative abundances of *Parasutterella* and *Odoribacter* are associated with cognitive and spatial learning impairments [42,56]. *Helicobacter* and *Odoribacter* are significantly increased in cognitive-altered db/db mice and in D-galactose-induced cognitive impairment mice [57,58]. Interestingly, supplementation with peptides derived from walnut in a model of aging induced by D-galactose injection improves cognitive impairment by regulating the composition of the microflora and notably preventing the increase in *Helicobacter* and *Odoribacter* [58].

It has been demonstrated that microbiota dysregulation is highly correlated with cognitive impairment and may influence social behavior and stress regulation [59,60,61,62]. Stress not only influences the composition of the gut microbiota, but the gut microbiota also impacts the stress response and brain neurochemistry [63,64]. Germ-free mice subjected to acute restraint stress exhibit an amplified reaction of the HPA axis, resulting in increased corticosterone levels. This dysfunction is rectified when germ-free mice are colonized with commensal bacteria [65]. In our study, aged mice had higher basal serum corticosterone levels and impaired HPA axis response to stressful stimuli compared with young mice. These findings confirm our previous study [31] and corroborate the results of Tronche et al. [66] reporting that the stress system of aged mice is not as controlled as that of young mice under normal physiological conditions and does not effectively respond to incoming stress compared with young mice. Moreover, the studies conducted by Knight and Durbin [67] and Puigoriol-Illamola et al. [68] have established a correlation between anxiety and memory capacity. They indicate that individuals with anxiety are more susceptible to experiencing memory deficits and that inhibiting the enzyme responsible for glucocorticoid synthesis can prevent cognitive impairment [67,68]. Our results indicate that fish hydrolysate supplementation restored basal corticosterone levels in aged mice and both supplementations decreased the stress reactivity. The Spearman’s rank correlation analysis revealed a negative correlation between the AUC of the corticosterone level and social behavior. Moreover, we also demonstrated that microbiota composition was highly correlated with social novelty preference suggesting that the fish hydrolysate supplementation enhanced social behavior by modulating the level of corticosterone and the microbiota profile.

Furthermore, gut microbiota also plays a role in shaping the host’s circadian rhythms that are also modified during aging [69,70]. A comparison between germ-free mice and specific pathogen-free mice has corroborated that microbiota significantly induce diurnal circadian rhythms within liver hepatocytes [71]. Our results suggest a decrease in the expression of two circadian genes, *Bmal1* and *Per1,* with aging. Few studies have investigated the expression of clock genes on learning and memory processes in relation to aging. However, *Bmal1* has been shown to play a significant role in aging-related pathology [72,73]. Indeed, *Bmal1*^−/−^ mice exhibit impairments on hippocampus-dependent cognitive tasks, including the Morris water maze [74]. In addition, it has been shown that reducing *Per1* mRNA levels in the hippocampus by 30% leads to impaired hippocampal learning in adult mice [75]. Conversely, when *Per1* is overexpressed in the hippocampus of aged mice, it prevents age-related hippocampal memory deficits, indicating that *Per1* plays a crucial and causal role in hippocampal memory processes. Taken together, these findings suggest that the upregulation of *Per1* following learning contributes to the subsequent formation of long-term memories in the hippocampus [76]. By restoring the expression of *Per1* and *Bmal1* genes to the same level as in adult mice, fish hydrolysate therefore appeared to be an interesting candidate for preventing circadian clock alterations during aging.

In addition, previous studies have highlighted the essential role of mitochondria in the communication between the gut and brain [77,78]. The modulation of mitochondria, and especially a decrease in the activity of mitochondrial electron transfer complexes, is also associated with behavioral dysfunctions during aging [79,80]. Defects in complex I are associated with several human neurological disorders [81]. Deficits in the respiratory chain contribute to increased ROS production, resulting in age-dependent memory impairments and behavioral dysfunctions [79,80,82,83]. In our study, aging decreased the expression of mitochondrial complex 1 genes ND1, ND2, ND5, and ND6 and fish hydrolysate supplementation restored their expression to the same level as in adult mice. In addition, we showed for the first time, a positive correlation between the level of the mitochondrial respiratory chain gene expression and the index of social novelty preference, suggesting that the increased expression of the mitochondrial respiratory chain gene expression induced by the fish hydrolysate supplementation may enhance social behavior.

Finally, the addition of n-3 LC-PUFAs to the fish hydrolysate did not have an additional effect compared to the fish hydrolysate alone. The fish hydrolysate contained mainly low-molecular-weight peptides (2.9 mg/d) and a small amount of n-3 LC-PUFAs (130 µg/d). Our result suggests that the effect observed was only due to low-molecular-weight peptides as reported in two studies on the effect of peptides on gut microbiota and cognitive impairment or that the amount of n-3 LC-PUFAs contained in the fish hydrolysate was sufficient to produce an additional effect and improve gut microbiota and social behavior. The beneficial role of n-3 LC-PUFAs on microbiota is poorly described and only in adults. The long-term administration of a high dose of EPA/DHA (250 mg/d for 5 weeks) in female rats leads to restoration of the normal Firmicutes/Bacteroidetes phyla ratio and decreases the abundance of proinflammatory Proteobacteria phylum [84]. Short-term n-3 LC-PUFA supplementation (990 mg EPA + 990 mg DHA for 7 days) modulates microbiota in a mucosal simulator of the human intestinal microbial ecosystem [85]. But the major effects of EPA and DHA seems to be associated with their anti-inflammatory properties and may act on gut permeability [86]. To be sure that the effect of n-3 LC-PUFA in the fish hydrolysate is not sex-dependent, a novel study should be conducted on female mice.

Consequently, fish hydrolysate can be considered as a multipotent actor to prevent cognitive and social alteration during aging via a direct pathway on the HPA axis through a benzodiazepine-like effect [87] that contributes to the prevention of social alteration, and/or via an indirect pathway through the microbiota. Indeed, microbiota may impact hippocampal-dependent memory and social behavior mechanisms through different pathways [88]. Microbiota may activate the vagal afferent nerves that promote hippocampal spatial memory. Microbiota may also impact the HPA axis through modulating the levels of corticosterone that modulate hippocampal memory. Moreover, microbiota is able to produce signaling molecules, such as short-chain fatty acids, that have strong effects on memory through enhancing BDNF associated with neurogenesis that is highly decreased during aging. Finally, microbiota may influence the circadian rhythms as shown in our study and others [89]. Microbiota may also play an important role in the homeostasis of oxidative molecules that induce mitochondrial dysfunction as shown in our study and prevent alteration of social interaction. These pathways can overlap and interact and hence have to be considered altogether to depict a precise picture of the effect of fish hydrolysate.

## 5. Conclusions

In this study, we are the first to demonstrate a positive impact of fish hydrolysate with low-molecular-weight peptides and n-3 LC-PUFAs on social behavior and to confirm its interesting results on cognitive functions, anxiety-like behavior, and stress response in aged mice. The beneficial effects induced by the hydrolysate supplementation on gut microbiota, corticosterone levels, circadian clock, and mitochondrial respiratory chain reinforced the innovative character of this active ingredient on the prevention of age-related impairments. Finally, the addition of n-3 LC-PUFAs to the existing fish hydrolysate did not have an additional effect compared to fish hydrolysate alone, suggesting that it did not act synergistically with the fish hydrolysate. Our results highlight a new target to treat age-related behavioral alterations by acting on gut microbiota. Moreover, with a view to preventive and personalized nutrition, fish hydrolysate appears as a good candidate for preventing the alteration of memory performance and social behavior during aging.

## Figures and Tables

**Table 1:** Composition of the control and hydrolysate-enriched diets.

Components	Percent (%)		
	Control Diet	Hydrolysate-Enriched Diet	Hydrolysate-Enriched Diet with EPA/DHA
Hydrochloric casein	18	18	18
Corn starch	45.9	45.69	45.69
Sucrose	24	24	24
Cellulose	2	2	2
Peanut Oil	5	5	4.91
Mineral Mix	4	4	4
Vitamin Mix	1	1	1
+DL methionine	0.1	0.1	0.1
+Vitamin A 5 UI/g	5 UI/g	5 UI/g	5 UI/g
Hydrolysate	0	0.21	0.21
EPA/DHA (4:1)	0	0	0.09

**Table 2:** Genes significantly modulated by age and/or supplementations in hippocampus.

Symbol	Name	Sequence (5′-3′)	Pathway
ND1-F	NADH dehydrogenase 1, mitochondrial	GAGTTCCCCTACCAATACCACACC	Mitochondrial respiratory chain
ND1-R		CGGCTCGTAAAGCTCCGAATAG	
ND2-F	NADH dehydrogenase 2, mitochondrial	TTCATAGGGGCATGAGGAGGAC	
ND2-R		GTGAGGGATGGGTTGTAAGGAAG	
ND5-F	NADH dehydrogenase 5, mitochondrial	GGAAGCATCTTTGCAGGATTTG	
ND5-R		CATGGTATTGTGAGGACTGGAATG	
ND6-F	NADH dehydrogenase 6, mitochondrial	TGGTTTGGGAGATTGGTTGATG	
ND6-R		TATTGCCGCTACCCCAATCC	
Bmal1-F	Basic helix-loop-helix ARNT like 1	CGCTACGAAGTCGATGGTTCAG	Circadian clock
Bmal1-R		TGTTAGCTGCGGGAAGGTTG	
Per1-F	Period circadian clock 1	TGTCCTGCTGCGTTGCAAAC	
Per1-R		TTGAGACCTGAACCTGCAGAGG	
SOD1-F	Superoxide dismutase 1	TTGGCCGTACAATGGTGGTC	Antioxidant defenses
SOD1-R		GCAATCCCAATCACTCCACAG	
APOE-F	Apolipoprotein E	TGCGAAGATGAAGGCTCTGTG	Microglial neurodegenerative phenotype
APOE-R		GGTTGGTTGCTTTGCCACTC	
mTrem2-F	Triggering receptor expressed on myeloid cells 2	TGCTGGCAAAGGAAAGGTGC	
mTrem2-R		ACATGACACCCTCAAGGACTGG	
mCd68-F	Cluster of Differentiation 68	TTCGGGCCATGTTTCTCTTG	
mCd68-R		ATTGTCGTCTGCGGGTGATG	
mClec7a-F	C-Type Lectin Domain Containing 7A	TGGGTGCCCTAGCATTTTGG	
mClec7a-R		TGATTCTGTGGGCTTGTGGTTC	
mLgals3-F	Galectin 3	TATCCTGCTGCTGGCCCTTATG	
mLgals3-R		TGCGTTGGGTTTCACTGTGC	
mCcl2-F	C-C Motif Chemokine Ligand 2	ACCAGCCAACTCTCACTGAAGC	
mCcl2-R		TGGGGCGTTAACTGCATCTG	
Acox1-F	Peroxisomal acyl-CoA oxidase	TCACTCGAAGCCAGCGTTAC	b-oxidation
Acox1-R		TTGAGGCCAACAGGTTCCAC	
BDNFIV-F	Brain Derived Neurotrophic Factor	CAGAGCAGCTGCCTTGATGTT	Neuroprotection
BDNFIV-R		GCCTTGTCCGTGGACGTTTA	
HSP90-F	Heat shock protein 90	TTGGTGGACACAGGCATTGG	Aging
HSP90-R		AATCCGACACCAAACTGCCC	
HSF1-F	Heat shock factor 1	TGGCCATGAAGCACGAGAAC	
HSF1-R		TTTGCTGCTGGGCATGCTTC	
Cmklr1-F	Chemerin Chemokine-Like Receptor 1	AGTCACGCGCAGTAACAGAC	Synthesis of oxylipins
Cmklr1-R		TCGTTGTAAGCGTCGTACTCC	
ALXFPR2-F	Formyl peptide receptor 2	CATGAAGTCTGTGATAAGGGATGG	
ALXFPR2-R		CATAAGGTAAGGAAGGCAGAAGTG	
12LOX-F	12-lipoxygenase	GCTGTTGCCACCATGAGATG	
12LOX-R		AACGGATGTGTGGAACGAGG	
Ptges-F	Prostaglandin E Synthase	ATCAAGATGTACGCGGTGGC	
Ptges-R		ATCCTCGGGGTTGGCAAAAG	
Ptgs1-F	Prostaglandin-Endoperoxide Synthase 1	ATCACCTGCGGCTCTTCAAG	
Ptgs1-R		ATCAACACGGACGCCTGTTC	

## Data Availability

Data is contained within the article or Supplementary Material.

## Supplementary Materials

The following supporting information can be downloaded at: https://www.mdpi.com/article/10.3390/foods12234199/s1, Figure S1: Preference for social novelty and memory performance correlate with (A) AUC of corticosterone. (B) Plasmatic level of Kynurenine. (C) Kynurenine/Tryptophan ratio. (D) Gene expression of complex 1 of the mitochondrial respiratory chain and (E) Gut microbiota composition.
