# Supplementary material for: Dietary Marine Hydrolysate Improves Memory Performance and Social Behavior through Gut Microbiota Remodeling during Aging
Source: Foods. 2023 Nov 21;12(23):4199. doi: 10.3390/foods12234199 (PMC10706458; doi:10.3390/foods12234199)
Supplement: Supplementary file 1 [file foods-12-04199-s001.zip › foods-2653267-supplementary.pdf]

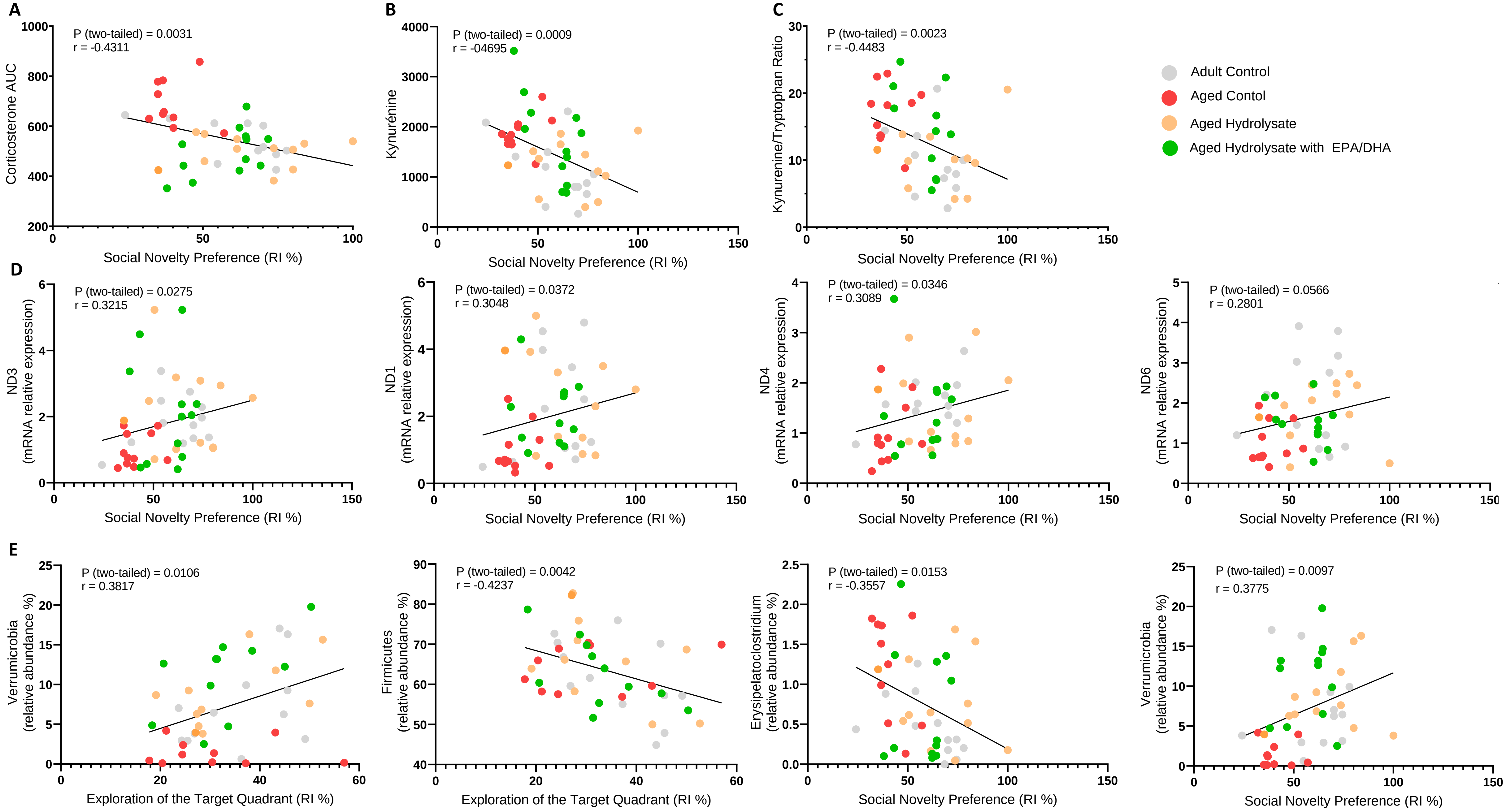

Supplementary Figure S1. Preference for social novelty and memory performance correlate with (A) AUC of corticosterone. (B) Plasmatic level of Kynurenine. (C) Kynurenine/Tryptophan ratio. (D) Gene expression of complex 1 of the mitochondrial respiratory chain and (E) Gut microbiota composition.
